# Effects of Prebiotic Phytocompound Administration in Gestational Diabetic Dams and Its Influence on Offspring Cognitive Outcomes

**DOI:** 10.3390/ijms26073140

**Published:** 2025-03-28

**Authors:** Gayathri Jagadeesan, Tushar K. Das, Jennifer M. Mendoza, Ghalya Alrousan, Maria P. Blasco-Conesa, Parimelazhagan Thangaraj, Bhanu Priya Ganesh

**Affiliations:** 1 Department of Neurology, The University of Texas McGovern Medical School, Houston, TX 77030, USA; gayathri.jagadeesan@uth.tmc.edu (G.J.); tushar.k.das@uth.tmc.edu (T.K.D.); jennifer.m.mendoza@uth.tmc.edu (J.M.M.); ghalya.alrousan@uth.tmc.edu (G.A.); maria.p.blascoconesa@uth.tmc.edu (M.P.B.-C.); 2 Bioprospecting Laboratory, Department of Botany, Bharathiar University, Coimbatore 641-046, Tamil Nadu, India; drparimel@gmail.com; 3 The University of Texas MD Anderson Cancer Center UTHealth Graduate School of Biomedical Sciences, Houston, TX 77030, USA

**Keywords:** gestational diabetes, gut microbiota, dysbiosis, phytocompounds, prebiotics, maternal health, cognition

## Abstract

Gestational diabetes mellitus (GD)-induced gut dysbiosis in pregnant mothers may increase the risk of cognitive impairment and neurological disorders in both the mother and offspring as they age. Restoring gut balance could improve cognitive outcomes for both. Despite advancements in GD treatment, side effects have increased, and long-term neurocognitive impacts on offspring born to GD mothers remain underexplored. This study uses a GD mouse model, inducing pancreatic dysfunction in 3-month-old pregnant C57BL/6J mice with Streptozotocin. The efficacy and mechanism of the prebiotic phytocompound green leaf extract (*Allmania nodiflora)* were assessed, with metformin as the standard. GD dams exhibited weight and glucose reduction, pancreatic IL-6 elevation, GLUT3 reduction, astroglia changes in the cerebral cortex, gut barrier impairment, cognitive impairment, and heightened anxiety compared to controls. Bacterial 16s rRNA sequencing revealed dysbiosis, with reduced *Erysipelotrichales* in GD dams compared to controls. Metformin lowered blood glucose levels but failed to rescue functional and behavioral phenotypes in both GD dams and offspring. Phytocompound treatment improved blood glucose, reduced pancreatic inflammation, improved gut barrier integrity, reversed dysbiosis, and enhanced brain health. It rescued behavioral deficits and improved cognitive outcomes in offspring, suggesting the prebiotic phytocompound may be a more effective therapeutic agent for GD in humans.

## 1. Introduction

Gestational diabetes mellitus (GD) develops during the second and third trimester of gestation and affects around 14% of all pregnancies worldwide [1]. GD affects up to 9% of pregnancies in the U.S. each year and occurs between 24 and 28 weeks of pregnancy [2]. GD poses significant short-term and long-term health risks to both the mothers and their offspring. In addition to the stress of a normal pregnancy, there is an increased risk of preterm birth and pre-eclampsia, leading to severe and prolonged maternal health issues. About 60% of women with a history of GD have a seven-fold increase in the risk of developing type-2 diabetes mellitus later in life [3]. Complications during pregnancy can also directly impact the neuronal health of the fetus [4]. The gut microbiome regulates insulin sensitivity [5], and GD affects the maternal microbiome, altering its composition and function, resulting in dysbiosis. Dysregulation of the microbiome in GD pregnancies has been linked to fetal neurodevelopmental disorders [6]. However, the impact of GD-associated dysbiosis on short- and long-term neurological outcomes is understudied in both mothers and offspring. There is a critical need to identify and dissect the mechanisms underlying the neurodevelopmental and neuroinflammatory consequences of post-GD dysbiosis in both mother and offspring, particularly as they age.

Treatment for GD is to reverse hyperglycemia and reduce the risk of adverse pregnancy outcomes. However, the standard therapeutic approaches for diabetes, including insulin therapy and oral hypoglycemic medication (metformin and glibenclamide), present significant challenges. While these interventions may be effective in controlling glucose levels, they carry risks, especially during pregnancy, with potential side effects [3]. Dietary interventions are known to have a beneficial impact with no side effects. Lately, there has been a developing interest in the potential therapeutic benefits of plant-derived phytocompounds in managing diabetes and its complications [7]. Specifically, diverse green leafy vegetables (GLVs) contain anti-diabetic compounds that help combat postprandial hyperglycemia and are effective in managing blood sugar levels. Notably, *Allmania nodiflora* (L.) R.Br. ex Wight, a tropical wild green leafy vegetable from the family *Amaranthaceae*, grows in fields as a weed in Asia, predominantly in the Western Ghats, India [8]. It has gained attention due to its anti-diabetic properties, but the mechanisms are not known. Given its traditional use and potential bioactivity, *Allmania nodiflora* was selected for further investigation in this study [8]. An increase in GLV intake may also directly influence the gut microbiota by providing phyto-fibers, which are known to be prebiotics that enhance beneficial microbes and their metabolites [9].

Streptozotocin (STZ), an antibiotic derived from streptomycin, exhibits a highly selective toxic effect on the islet β cells of experimental animals, resulting in insufficient insulin secretion and elevated blood glucose levels [10]. Consequently, it is one of the most commonly used drugs for inducing diabetes in animal models. In recent years, numerous studies have demonstrated that intraperitoneal injection of STZ is one of the ideal methods for establishing experimental GD animal models. This approach is simple, effective, cost-efficient, time-saving, and highly reproducible, making it widely used in GD research [11,12,13]. To investigate the impact of GD on the short- and long-term cognitive health of both dams and offspring, 3-month-old virgin C57/BL6 mice were used, which were bred with age-matched male mice. After a positive pregnancy was identified, STZ was administered to the animals from gestational Day 7 until Day 12. The glucose tolerance test showed a significant increase in glucose levels after the STZ challenge, causing the gestational mice to become diabetic. Two controls were used in the experiment: the treatment control, which was administered metformin [14,15,16,17] from Day 13 to Day 18, and the negative control, a vehicle-treated healthy pregnant group. The experimental groups were challenged with STZ from Day 7 to Day 12, followed by treatment with phytocompound from Day 13 to Day 18 of the gestational period.

This study aims to explore the beneficial effects of a bioactive phytocompound (*A. nodiflora*) extract containing phyto-fibers [8] on maternal glucose levels and cognitive outcomes in dams that were induced with gestational diabetes during their pregnancy and their respective offspring. This study aims to investigate whether supplementing with phytocompounds rich in phyto-fibers, antioxidants, polyphenols, and other bioactive compounds can prevent dysbiosis and reduce glucose levels in diabetic pregnant mice during gestation. A comprehensive study has been performed to examine the impact of these phytocompounds on various physiological parameters, aiming to understand their efficacy and mechanism of action in gestational diabetic dams. Additionally, behavioral studies were conducted on both dams prior to delivery and on pups post-birth to evaluate the cognitive outcomes and unravel the connection between metabolic health and behavior in gestational diabetes. The murine model of GD could provide valuable insights into the efficacy and safety of *A. nodiflora* as a potential dietary intervention for pregnant women, a novel approach to improve maternal and fetal neurocognitive outcomes. However, not many reports are available on these approaches for investigating and preventing anti-diabetic activity during pregnancy. This study broadens the knowledge of therapeutic approaches and also integrates traditional medicine with modern healthcare practices. The results from this study could contribute to the development of a novel therapeutic agent combining prebiotic and probiotic properties, offering a natural, safer, and more sustainable alternative to conventional treatments for combating GD [18,19].

## 2. Results

### 2.1. GD in Dams Leads to Significantly Higher Blood Glucose Levels and Reduced Body Weight, and Both Were Reversed with Phytocompound Therapy

**Figure 1:**
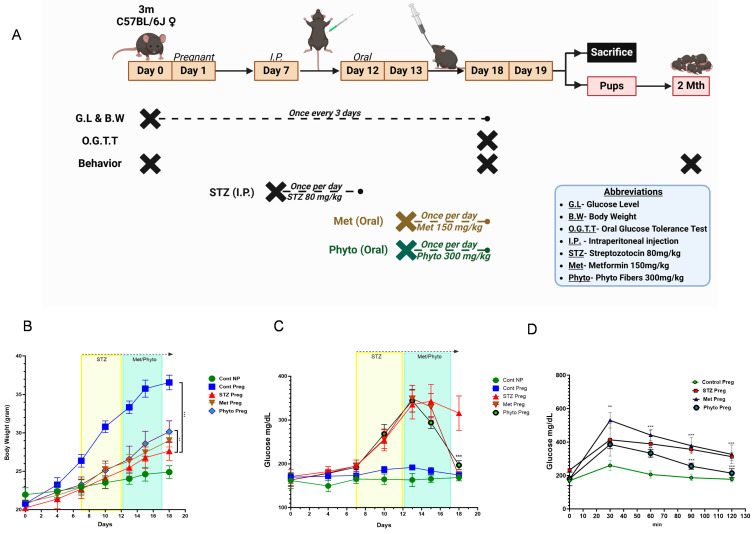
(**A**) Experimental design showing the flowchart of the gestational period, STZ challenge to induce diabetes, and treatment period (metformin or phytocompound), followed by behavioral assessment and organ harvesting. (**B**) Body weight was measured in pregnant mice (identified by a positive vaginal plug) every three days until sacrifice. Weights were recorded in grams (g). (**C**) Glucose level measurements were taken every three days from Day 1 of gestation until gestational Day 18. Quantification of glucose levels is shown in mg dl^−1^. (**D**) An oral glucose tolerance test (OGTT) was conducted on Day 18, with measurements taken at 30, 60, 90, and 120 min post-administration. The results are presented in mg/dL relative to the recorded time intervals. Tukey’s two-way ANOVA with multiple comparisons was used for statistical analyses (**B**,**C**). *n* = 15–18 per group. All data (with error bars) are presented as the mean ± SEM. * *p* < 0.05, ** *p* < 0.01, *** *p* < 0.001. All data points presented are biological replicates. Cont NP—healthy non-pregnant control; Met Preg—metformin therapy; STZ Preg—Streptozotocin challenged; Phyto Preg—phytocompound therapy; Preg—pregnant.

Blood glucose levels were analyzed using a glucose glucometer, and body weight was recorded using a scale, with both measurements taken every three days throughout the study. The oral glucose tolerance test (OGTT) for animals in all groups was recorded once at the end of the experimental week. The initial body weights and blood glucose levels (Day 0) of all animals, prior to mating, were noted. Subsequent measurements were taken during pregnancy and after treatment (Figure 1A). In the non-pregnant control group (NPC), there was a gradual increase in body weight from 21.9 ± 0.90 g to 24.8 ± 0.82 g throughout the experiment (Figure 1B), while glucose levels were maintained between 149.6 ± 13.01 mg/dL and 168.8 ± 7.5 mg/dL. The pregnant control group (PC) showed a notable increase in body weight following pregnancy between 22.3 ± 0.83 g and 29.2 ± 0.95 g, stemming from typical gestational weight gain (Figure 1B). In contrast, the gestational diabetic group (GD), which received Streptozotocin (STZ) on Day 7 to induce diabetes, showed reduced weight gain throughout pregnancy due to elevated blood glucose levels. Between Days 7 and 12, body weight in the GD group was compared to that of the NPC group (Figure 1B). During the course of STZ administration, it was observed that some mice experienced pregnancy loss due to gestational diabetes, resulting in a reduced pup litter size, while others remained pregnant and gained weight. The metformin treatment group (MT) and phytocompound treatment group (PT) exhibited improved weight gain (29.0 ± 0.84 g and 29.4 ± 1.41 g, respectively) compared to the GD group (27.6 ± 1.21 g) (Figure 1B). Notably, the PT group exhibited better weight gain than the MT group, implying that phytocompound treatment in diabetic mice led to significant weight gain during gestation, providing sufficient nutrients for maternal weight gain and fetal development.

All female animals used in the study measured by glucose glucometer showed normal blood glucose levels between 169.6 ± 7.79 mg/dL and 172 ± 9.30 mg/dL before pregnancy. After pregnancy, there was a moderate increase in blood glucose, reaching 192 ± 5.70 mg/dL on Day 13; although, this was not within the diabetic range found in healthy pregnant control mice (PC group). By the end of the pregnancy on Day 18, glucose levels returned to the normal range, measuring 174.6 ± 5.17 mg/dL (Figure 1C). The GD group that received STZ from Day 7 until Day 12 during pregnancy showed an increase in blood glucose levels by Day 10, reached 249.2 ± 27.98 mg/dL, and peaked at Day 15 (361.8 ± 29.15 mg/dL). Both the metformin- (MT) and phytocompound-treated (PT) groups also experienced high glucose spikes of 357 ± 34.37 mg/dL and 355.2 ± 24.46 mg/dL, respectively, during the STZ treatment phase measured on Day 13 (Figure 1C). However, following metformin treatment from Day 13 to Day 18, the MT group showed no initial changes in glucose levels until Day 15 but showed a marked drop in glucose levels to 177.4 ± 8.73 mg/dL on Day 18 from the pre-treatment time point of 336.8 ± 35.08 mg/dL (Figure 1C). In contrast, the phytocompound-treated (PT) group exhibited a gradual decrease in glucose levels during the treatment measured on Day 13 from 355.2 ± 24.46 mg/dL to 295.6 ± 17.00 mg/dL on Day 16, eventually reaching 201.6 ± 8.23 mg/dL on Day 18 (Figure 1C). The accelerated glucose reduction caused by metformin may lead to hypoglycemia, and, in addition, it has known side effects like abdominal pain and diarrhea. Also, metformin treatment did not prevent weight loss in the pregnant dams, whereas the phytocompound treatment exhibited steady glucose reduction with gradual weight gain.

The oral glucose tolerance test (OGTT) primarily measures processed glucose levels in the body, particularly to assess insulin resistance, which can lead to insulin deficiency and the failure of glucose homeostasis. After a 4 h fast, the GD group showed a peak glucose level of 260.25 ± 11.23 mg/dL, which was higher than that of the control and treatment groups (PC—169.25 ± 9.46 mg/dL; MT—180 ± 4.39 mg/dL; PT—180.25 ± 3.94 mg/dL) (Figure 1D). Following the glucose solution gavage, blood glucose was measured at 30 min intervals, with all groups reaching peak glucose levels between 30 and 60 min. The GD group showed significantly increased blood glucose at 60 min (414.5 ± 6.09 mg/dL) and did not return to the normal range even after an hour (312.75 ± 2.43 mg/dL at 120 min). Metformin treatment in the MT group resulted in lower blood glucose, although it did not return glucose to normal levels. In contrast, the PT group showed a significant glucose reduction from 333.5 ± 2.29 mg/dL to 255.75 ± 1.85 mg/dL at 90 min, nearly reaching the normal level of 214.25 ± 2.63 mg/dL. Compared to metformin, the phytocompounds effectively lowered blood glucose, almost reaching control levels. The sustained high glucose level in the GD group at 120 min indicates insulin deficiency and insulin resistance during pregnancy (Figure 1D).

### 2.2. GD Dams Showed Significantly Elevated Levels of the Pro-Inflammatory Cytokine Interleukin 6 (IL-6) and Glucose Transporter 2 (GLUT2) Expression in the Pancreas During Pregnancy

**Figure 2:**
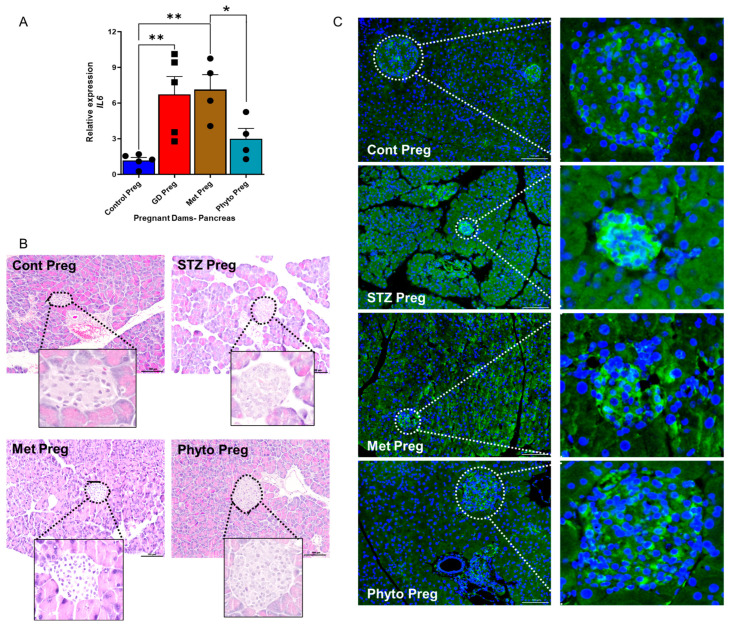
Pancreatic physiology and anatomy during gestational period in animals with and without diabetes. (**A**) Interleukin-6 (IL-6) expression in the pancreas was obtained from pregnant dams on Day 19 post-pregnancy. Tukey’s one-way ANOVA with multiple comparisons was used for statistical analyses. *n* = 4–5 animals per group. All data (with error bars) are presented as the mean ± SEM. * *p* < 0.05, ** *p* < 0.01. All data points presented are based on individual animals. (**B**) H and E staining of the pancreas showing islets of Langerhans, with dotted lines indicating magnified islets. (**C**) Glucose Transporter 2 (GLUT2) expression by fluorescence immunohistochemistry in the pancreas of dams on Day 19, with dotted lines indicating magnified islets. Green indicates GLUT2, and blue denotes nuclei (DAPI). All images were taken at 20× magnification, with the scale bar representing 100 µm. Cont Preg—control pregnant; Met Preg—Metformin; STZ Preg—Streptozotocin challenged; Phyto Preg—phytocompound.

Pancreas samples from GD dams were obtained on Day 19 of pregnancy and used for analysis. The mRNA transcriptomics data showed a significant increase in IL-6 transcriptomic levels within the pancreas of GD dams compared to healthy control dams (Figure 2A). Furthermore, IL-6 was downregulated with phytocompound treatment in the pancreas of GD animals but had no significant changes when GD animals were treated with metformin (Figure 2A). Interestingly, the phytocompound-treated GD pancreas showed the least production of IL-6 compared to other GD groups (Figure 2A). IL-4 and IFN-γ were not significantly different. Hematoxylin and eosin-stained pancreatic sections revealed a decrease in islet size in the GD group compared to healthy control animals (Figure 2B). No major morphological differences were observed between the metformin- and phytocompound-treated groups and control pregnant animals. Glucose Transporter 2 (GLUT2) was measured in the pancreas of dams on Day 19. Analysis revealed reduced GLUT2 expression due to the loss of beta cells in GD animals treated only with STZ, compared to healthy pregnant dams (Figure 2C). Metformin did improve the GLUT2 expression in the pancreas, not only in islets of Langerhans but also around them, as confirmed by confocal microscopy analysis of the pancreas with fluorescently labeled anti-GLUT2 staining (Figure 2C).

### 2.3. GD in Dams During Pregnancy Led to the Loss of Beneficial Gut Microbiota and Impaired Gut Integrity, as Indicated by 16s RNA Sequencing and Reduced E-Cadherin Levels

**Figure 3:**
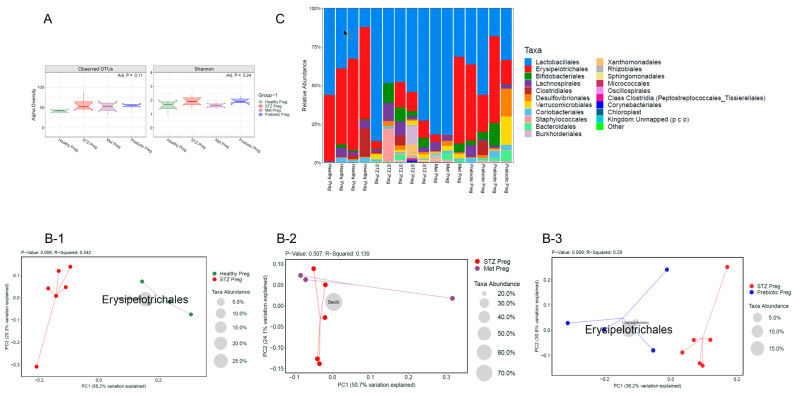
Compositional differences in cecal gut microbiota were analyzed by 16S rRNA sequencing and qPCR of intestinal luminal content. (**A**) Visualization of alpha diversity, or differences within-sample diversity, measured by both Observed OTUs and Shannon Diversity, respectively. (**B-1**) Visualization of beta-diversity, or between-samples diversity, using weighted UniFrac distances by principal coordinate analysis (PCoA) shows a clustering effect by strain between healthy pregnant dams and STZ-challenged GD pregnant dams on Day 19 post-pregnancy. The highest diversity was in the class *Erysipelotrichales*, and the second highest was in the class *Coriobacteriales*. (**B-2**) Visualization of beta-diversity, or between-samples diversity, using weighted UniFrac distances by principal coordinate analysis (PCoA) shows a clustering effect by strain between STZ-challenged GD pregnant dams (STZ Preg) and metformin-treated GD pregnant dams (Met Preg) on Day 19 post-pregnancy, along with corresponding family-level bacterial distribution. (**B-3**) Visualization of beta-diversity, or between-samples diversity, using weighted UniFrac distances by principal coordinate analysis (PCoA) shows a clustering effect by strain between STZ-challenged GD pregnant dams and phytocompound-treated GD pregnant dams (Prebiotic preg) on Day 19 post-pregnancy, along with corresponding family-level bacterial distribution. The highest diversity was in the class *Erysipelotrichales*, and the second highest was in the class *Coriobacteriales*. (**C**) Visualization of a graph showing the phylogenetic bacterial class between different groups. *n* = 3–6. Each dot represents an individual animal.

**Figure 4:**
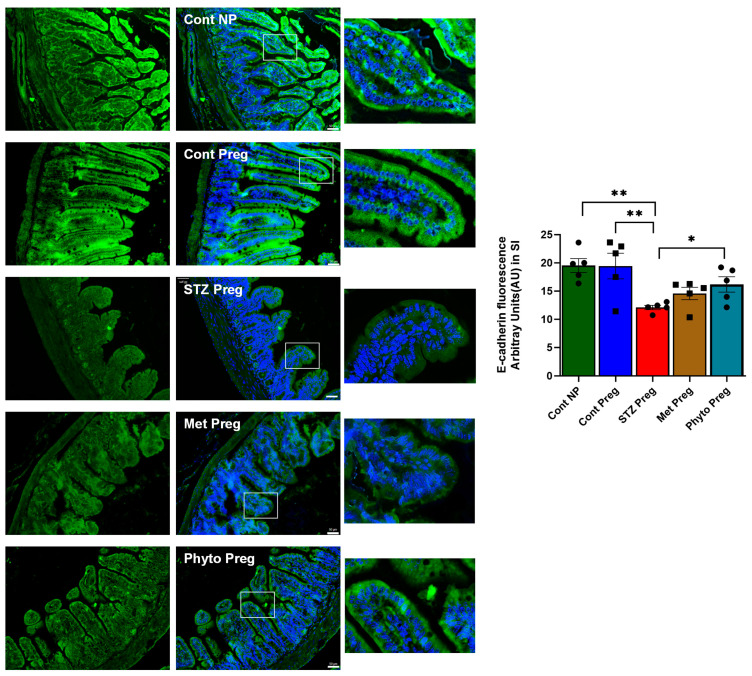
Gut barrier integrity was measured by E-cadherin expression levels in the ileum. Fluorescence IHC was used to measure E-cadherin expression levels in the ileum of mice obtained from all five groups. Imaged at 20× magnification, with the scale bar representing 50 µm. Data are quantified by targeting fluorescence green intensity using ImageJ. One-way ANOVA with Tukey’s multiple comparisons was used for statistical analyses. *n* = 5 animals per group. All data are presented as mean ± SEM (with error bars), and statistical significance is indicated as * *p* < 0.05, ** *p* < 0.01. Cont Preg—control pregnant; Met Preg—metformin therapy; STZ Preg—Streptozotocin challenged; Phyto Preg—phytocompound therapy.

Gestational diabetes was found to cause dysbiosis (a loss in the beneficial bacterial population compared to healthy control groups) [20,21] in pregnant dams during gestation. As expected, no significant differences were observed in the alpha diversity (measures within sample diversity), and no differences were found between the operational taxonomic units (OTUs) (Figure 3A). The weighted UniFrac distances, determined by principal coordinate analysis, indicated a significant shift in the beta or inter-sample diversity in pregnant dams with GD compared to healthy pregnant control dams, analyzed on Day 19 of the gestational period (Figure 3B). It was found that metformin treatment did not rescue the dams in the MT group from dysbiosis caused by gestational diabetes and showed no significant changes in beta diversity, as measured by the beta diversity plot, compared to the dams in the GD group (Figure 3B,C). However, phytocompound treatment groups showed a significant shift in bacterial diversity that resembled healthy pregnant groups with an increase in the class *Erysipelotrichales* (Figure 3B,C). It is proposed that the changes in bacterial diversity due to phytocompound treatment in gestational diabetic dams may improve gut integrity by enhancing epithelial tight junction expression. The gut integrity was measured by E-cadherin levels in the gut epithelium [22]. The data revealed that GD dams showed significantly reduced ileal E-cadherin expression levels compared to healthy pregnant control dams (Figure 4). Interestingly, the metformin-treated (MT) groups did not show improved E-cadherin levels, whereas the phytocompound-treated (PT) groups showed a moderate increase in E-cadherin levels compared to the GD groups (Figure 4). A moderate increase in E-cadherin expression was observed, as measured by f-IHC, and significant anatomical changes were noted in the intestinal villi between the healthy control groups and the GD and metformin-treated groups (Figure 4).

### 2.4. GD Caused an Anxiety Phenotype with Cognitive Impairment in Both Dams and Offspring Born to GD Dams, Rescued by Phytocompound Treatment

**Figure 5:**
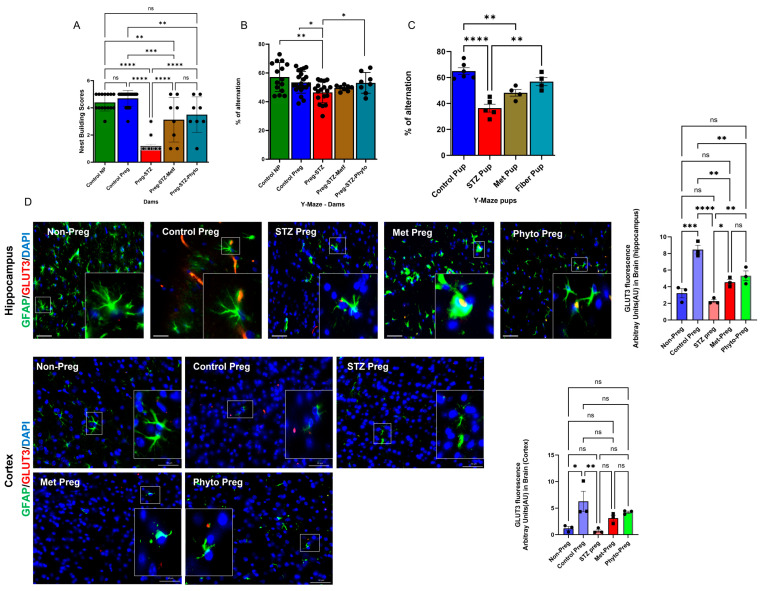
Behavioral analysis was measured in dams and offspring to evaluate cognitive function. (**A**) Nest-building score analysis was used to evaluate anxiety and depressive phenotype in dams with and without GD. (**B**) Cognitive impairment measured by Y-maze behavioral test in gestational diabetic dams. (**C**) Cognitive impairment measured by Y-maze behavioral test in offspring (at 2 months after birth) born to gestational diabetic dams. One-way ANOVA with Tukey’s multiple comparison test or Dunn’s multiple comparison test. *n* = 4–18 animals per group. All data are presented as mean ± SEM (with error bars), and statistical significance is indicated as * *p* < 0.05, ** *p* < 0.01, *** *p* < 0.001, **** *p* < 0.0001, ns means not significant. Each dot represents an individual animal. Cont NP—healthy non-pregnant control; Control preg—healthy pregnant; STZ—Streptozotocin challenged; Met—metformin therapy; Phyto—phytocompound therapy; Fiber—pups born from phytocompound treated dams. (**D**) Brain fluorescence IHC from dams to evaluate Glucose Transporter 3 (GLUT3) with astrocyte morphology. The brain hippocampus and cortex show astrocyte-specific marker GFAP in green and GLUT3 marker in red signal. Imaged at 20× magnification, with the scale bar representing 50 µm. GLUT3 expression levels are quantified using ImageJ. One-way ANOVA with Tukey’s multiple comparisons was used for statistical analyses. *n* = 3 animals per group. All data are presented as mean ± SEM (with error bars), and statistical significance is indicated as * *p* < 0.05, ** *p* < 0.01, *** *p* < 0.001, **** *p* < 0.0001, ns means not significant. Each dot represents an individual animal. Non-Preg—healthy non-pregnant control; Control Preg—healthy pregnant; STZ preg—Streptozotocin challenged; Met-Preg—metformin therapy; Phyto-Preg—phytocompound therapy.

Most importantly, a significant anxiety phenotype was observed in the GD groups, as evidenced by altered nest-building scores [23]. These scores were significantly different in the GD (STZ pregnant) groups compared to the healthy pregnant control (PC) groups on Day 18 of gestation (pre-delivery) (Figure 5A). Overall, phytocompound treatment (PT group) resulted in no significant difference in nest building scores compared to the MT group in GD mice during gestational period Day 18 (Figure 5A).

GD in dams during pregnancy, as well as in their offspring 2 months after birth, showed cognitive impairment (CI). The Y-maze test was used to assess spatial and working memory as indicators of cognitive impairment [24]. The Y-maze analysis revealed a poor memory phenotype, indicating cognitive impairment in GD group dams on Day 18 of their gestational period, compared to healthy pregnant dams (Figure 5B). Phytocompound treatment (PT) groups demonstrated a higher percentage of alteration, indicating better cognitive outcomes compared to the metformin treatment (MT) groups (Figure 5B). This underscores the effectiveness of phytocompound treatment over metformin treatment in GD dams during pregnancy.

The offspring from all four groups were aged until 2 months post-weaning and subjected to a cognitive behavioral test. The test revealed that the offspring born to GD dams exhibited significant cognitive impairment compared to those offspring born to healthy dams (Figure 5C). Notably, metformin treatment during pregnancy in GD dams failed to rescue the cognitive impairment observed in their offspring (Figure 5C). Interestingly, phytocompound administration in gestational diabetic dams (PT pregnant group) during pregnancy protected the offsprings’ neurocognition, resulting in improved cognitive outcomes at 2 months post birth, as measured by the Y-maze test (Figure 5C) compared to STZ pregnant or MT pregnant groups. While metformin therapy controlled the glucose spikes during pregnancy in GD dams, it did not prevent cognitive impairment in their offspring, whereas phytocompound therapy not only controlled glucose spikes but also improved the neurocognitive outcomes of offspring born to GD dams (Figure 5C).

### 2.5. Glucose Transporter 3 (GLUT3) in the Brain Was Significantly Altered Due to Gestational Diabetes in Pregnant Dams

GLUT3 and glial fibrillary acidic protein (GFAP) expression were measured in the brain sections, specifically in the hippocampus and cortical regions. GFAP denotes astroglia within the brain [25]. A fluorescently labeled antibody was used to measure expression levels using ImageJ from confocal microscopy images. A significant increase in GLUT3 expression was observed in both the hippocampus and cortex of healthy pregnant dams’ brains compared to non-pregnant mice brains (Figure 5D). The STZ challenge caused a significant decline in GLUT3 receptors in the dams’ brains. GLUT3 is a glucose transporter that is predominantly expressed in neurons for transporting glucose into the brain. Additionally, a significant reduction in GFAP-expressing astrocytes was observed with the progression of gestational diabetes in the STZ groups (Figure 5D). Both metformin and phytocompound administration rescued the GD brain from GLUT3 loss (Figure 5D). However, the phytocompound treatment in GD dams showed better protection against diabetes-induced inflammation than metformin-treated GD mice.

## 3. Discussion

Pregnancy is a significant and transformative period involving complex physical, emotional, and physiological adjustments for the mother as she nurtures and brings a new life into the world. Almost every endocrine tissue undergoes adaptive changes during normal pregnancy to maintain the metabolic state of the woman, aimed at survival and fetal growth [26,27]. During pregnancy, insulinemia progressively rises while insulin action at the hepatic level decreases, resulting in increasing blood sugar. During early gestation, insulin resistance helps to store glucose in adipose tissue to meet fetal demands [28]. As pregnancy progresses, hormonal changes cause prolonged insulin resistance, resulting in elevated blood glucose levels, which are transported to the fetus [29]. After delivery, maternal insulin sensitivity returns to pre-pregnancy levels. However, these metabolic adaptations do not occur adequately in all pregnancies, leading to gestational diabetes mellitus [30,31].

Treatment for gestational diabetes (GD) aims to reverse hyperglycemia and reduce the risk of adverse pregnancy outcomes. However, standard therapeutic approaches for diabetes, including insulin therapy and oral hypoglycemic medicines (metformin and glibenclamide), present significant challenges. Although these treatments can control glucose levels, they carry risks, especially during pregnancy, with potential side effects such as weight gain, diarrhea, renal failure, gastrointestinal disturbances, risk of hypoglycemia, drug resistance, and increased healthcare costs [3]. Metformin is commonly used to manage type 2 diabetes by reducing blood glucose levels through mechanisms such as reducing hepatic gluconeogenesis and enhancing insulin sensitivity [32,33]. Recent research has explored its potential neuroprotective effects in type 2 diabetes, although the exact mechanism remains unclear. These mechanisms may involve the modulation of mitochondrial function, a reduction in neuroinflammation, and changes in brain glucose metabolism [34]. While metformin is effective in managing type 2 diabetes, its use in GD and its potential effects on the central nervous system (CNS) remain elusive [35]. Metformin crosses the placenta and is used when insulin therapy or lifestyle modification are insufficient [35]. Despite the benefits of controlling maternal blood glucose, metformin is associated with certain risks due to its ability to alter the gut microbiota and cause gastrointestinal side effects [35,36], which may lead to dysbiosis, a condition linked to changes in neurocognitive function [37]. This study aims to further explore its potential to modulate neurocognitive outcomes in GD, highlighting the need for alternative therapies.

To achieve this, this study utilized a phytocompound extract from *A. nodiflora*, which contains plant metabolites and phyto-fibers, as a prebiotic therapy for GD dams during pregnancy. Plant fibers are known to change the gut microbiota composition by providing substrates and can modulate microbial metabolite production in the gut lumen. The data showed a significant reduction in blood glucose levels in GD dams treated orally with phytocompounds. Offspring born to the phytocompound-treated GD dams showed significantly improved cognitive outcomes and resembled the offspring born to healthy pregnant dams. Recent research on GD-associated maternal–child cohort studies has shown increased oxidative stress and inflammation during maternal diabetes, leading to elevated microglial activation. This pathologically modulates normal brain development and results in neurodevelopmental disorders, particularly intellectual disability, motor dysfunction, lower cognitive functioning, anxiety, schizophrenia, depression, and autism spectrum disorders [38]. In this study, GD significantly impaired cognitive dysfunction in both the dams and their offspring, with the offspring showing persistent cognitive deficits two months after birth. Although metformin treatment controlled glucose spikes in GD dams, no beneficial impact on the cognitive behavior of offspring born to metformin-treated GD dams was observed. The data show that dietary modifications can prevent these long-term deficits without any side effects by improving cognitive outcomes in offspring. This effect is likely due to changes in gut microbiota diversity.

The microbiota forms an intricate ecosystem with the host and adapts to the constantly fluctuating physiology of the host [39]. A large body of evidence has shown that microbiome imbalance, referred to as dysbiosis (a pathological microbiota composition when compared with healthy control microbiota composition) [20,21], affects the maternal metabolic profile, contributing to pregnancy complications that lead to compromised neonatal health [40]. GD mothers develop abnormal glucose intolerance, which is diagnosed between 24 and 28 weeks of gestation when the oral glucose tolerance test is performed. As a result of this glucose intolerance, maternal blood has a higher concentration of glucose than that in healthy mothers, leading to maternal metabolic derangements, including diabetes [41]. Evidence suggests that low-sugar, low-carbohydrate/high-fiber food has positive effects on glucose levels and helps protect GD mothers from high glucose spikes during pregnancy [42]. Fiber is known to act as a prebiotic, promoting the colonization of healthy microbiota in a diseased gut [9]. The gut microbiome is crucial for fetal development, especially neuronal and glial development and its maturation. A healthy maternal microbiome produces beneficial small molecules (bacterial-derived metabolites) that are known to enhance peripheral and brain immunity [43]. Pregnant and lactating mothers typically have a strong colonization of *Bifidobacteria* and *Lactobacillus* in their gut microbiome [44,45,46], which is important for the healthy development of their infant’s gut microbiota [44,47]. *Bifidobacterium* and *Bacteroides* appear as early keystone organisms, directing microbiota development and consistently predicting positive health outcomes [48]. Dysbiosis due to GD during pregnancy can lead to dysregulated immune activation, causing systemic inflammation, and has been linked to neurodevelopmental and psychiatric disorders caused by atypical brain development in offspring [6]. A comparative study between germ-free and specific-pathogen-free mice showed that gut microbial metabolites can cross through the placenta to the fetal compartment, confirming that gut microbes can affect fetal development [49]. Microbial dysbiosis has been found in the maternal gut microbiome of GD pregnancies [6]. The intestinal microbiota has been shown to modulate insulin resistance and the body’s inflammatory response [50,51]. Germ-free dams colonized with Bifidobacterium sp. during pregnancy led to increased neuronal synapse and glial development in the offspring [52]. In line with these studies, significant beta-diversity changes were observed in GD dams challenged with STZ, compared to vehicle-controlled health dams. Metformin treatment to control glucose levels did not have any significant impact on bacterial composition; in other words, the microbiota composition resembled that of GD dams. However, phytocompound-treated dams showed significant changes in gut microbiota diversity compared to GD dams, resembling the microbiota composition of healthy control dams. Specifically, the class *Erysipelotrichales* [53] was significantly increased with phytocompound therapy, as seen in healthy control dams. While species within the *Erysipelotrichales* class have been linked to adverse health outcomes [54], it is a diverse group, and specific species or strains within this class have been shown to have different impacts on metabolic health. The current study examines the increase in *Erysipelotrichales* class abundance, with observations showing that its abundance is higher in the microbiota of healthy pregnant controls and reduced in STZ-treated GD groups. In line with this finding, a recent study has also reported that the *Erysipelotrichales* class is decreased in infants born to gestationally diabetic mothers [55]. Another study showed that the family *Erysipelotrichaceae* (which belongs to the class *Erysipelotrichales*) was significantly lower in pregnant women diagnosed with gestational diabetes [56]. The phytocompound treatment reversed the GD-induced dysbiosis. The gut microbiota composition in phytocompound-treated groups resembled the healthy pregnant control groups. This shift in microbiota composition following phytocompound administration may influence the production of short-chain fatty acids, tryptophan metabolites, and other beneficial products. In the future, metabolomics studies will be conducted using GD animal models to investigate the beneficial impact of phytocompound administration on maternal and fetal health outcomes.

**Figure 6:**
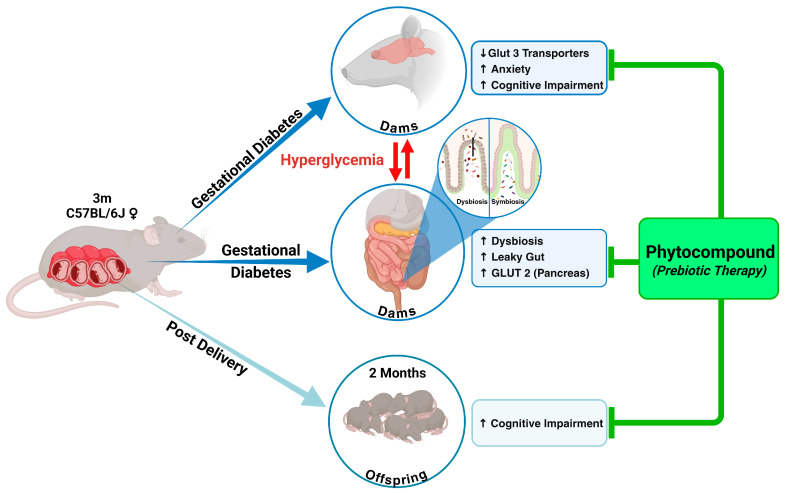
Schematic representation of the beneficial effects of phytocompound treatment on the health of gestational diabetic (GD) dams and their offspring. GD was associated with elevated blood glucose levels, impaired gut integrity, dysbiosis, and cognitive deficits in both the mother’s and offspring’s health. Treatment with phytocompounds from *Allmania nodiflora* decreased glucose levels, restored gut integrity, reversed dysbiosis, and improved cognitive performance in spatial and working memory in both GD dams and their offspring. In this figure, ↑ means elevation, and ↓ means reduction. Figure 6 was created in BioRender.com (https://BioRender.com/n36d528, accessed on 12 March 2025).

The study findings highlight the detrimental impact of gestational diabetes mellitus (GD) on maternal metabolic health, gut integrity, and neurocognitive outcomes in offspring (Figure 6). GD was associated with elevated blood glucose levels, reduced maternal weight gain, and impaired gut barrier integrity marked by E-cadherin in the intestinal epithelium. Behavioral analyses further revealed cognitive impairments and anxiety phenotypes in both GD dams and their offspring, with long-term consequences. While conventional metformin treatment-controlled glucose spikes effectively [14,15,16,17], it failed to prevent cognitive deficits in offspring and did not restore gut barrier integrity. In contrast, phytocompound treatment from *Allmania nodiflora* exhibited promising outcomes; it not only improved maternal weight gain and glucose regulation but also restored gut barrier integrity by enhancing E-cadherin expression. Moreover, phytocompound-treated dams and their offspring showed better cognitive outcomes, as evidenced by improved behavioral performances in Y-maze and nest-building tests. A limitation of the current study is the inability to fully replicate the intricate metabolic processes observed in humans due to the difference between animal models and human physiology. Future studies may consider utilizing alternative animal models that more closely mimic human metabolic conditions, including diet influences, to enhance these findings. As this study is not a long-term investigation, further research will explore the mechanisms and their effects on offspring in the long-term. This study primarily focuses on class-level microbiota changes observed in healthy control groups and gestational diabetic mothers using 16S rRNA sequencing. To further investigate strain-level difference and metabolic byproducts, additional techniques such as shotgun metagenomics and metabolomics will be utilized to enhance these findings. Our future experiments will utilize these techniques to further investigate the role of gestational diabetes in maternal and offspring long-term cognitive health.

## 4. Materials and Methods

### 4.1. Plant Extract Preparation

The phytocompounds were prepared from the extract of the green leafy vegetable *Allmania nodiflora.* The leaves were washed to remove dirt, and the healthy leaves were separated and rinsed with distilled water. The washed leaves were sliced and ground well using a mechanical blender (Sujata Dynamix-810W; Delhi, India) and boiled with an equal amount of water (1:1 *w*/*v*) in a heating mantle (Sigma soxhlet mantle, Chennai, India) at 70–80 °C for 15–20 min with stirring at regular intervals. Then, the mixture was allowed to cool at room temperature, and the extract was filtered using a muslin cloth; the procedure was repeated twice to extract the maximal amount of phytocompounds. The extract was condensed using a rotary vacuum evaporator (Sigma soxhlet mantle, Chennai, India) and dried at 50 °C in a hot air oven (New lab instruments, Chennai, India). The condensed and dried extract was stored at—20 °C for further analysis. The remaining plant material was stored at −80°C. This ensures that the same plant material can be used in future experiments, facilitating the reproducibility and validation of the results. The extensive process of plant collection and extraction was described in an earlier study [8].

### 4.2. Animals and Study Design

C57BL/6 (wild type) 6–8-week-old female mice were purchased from the Jackson Laboratory and acclimatized for a week. Then, the animals were housed up to 5 per cage in standard facilities (ventilated individually; changed weekly under a workstation with HEPA filter) with a 12 h light/dark schedule (from 7 am) in a temperature- (21.7–22.8 °C) and humidity-controlled (40–60 RH) vivarium. The animals had ad libitum access to food (LabDiet 5053 and 5058 irradiated pellets) and filtered tap water with a pH of 6–8 (not acidified nor chlorinated).

The female mice (10 weeks old) from all five groups were in the estrus stage at the time of mating. Each female was paired with a single male mouse (two females per cage with one male). After confirming gestation (Gestation Day 0) through the presence of a copulatory plug, the female mice were housed separately [57]. To induce diabetes, all the pregnant mice at gestation Day 7 were injected with a freshly prepared Streptozotocin solution (STZ, 80 mg/kg) (Sigma Aldrich, St. Louis, MO, USA) in citrate buffer for 5 days. Then, the blood glucose level was tested by taking a blood drop from the tail tip and measured using an Accu-Check active glucose glucometer kit (Roche, Indianapolis, IN, USA). Mice with a glucose level > 190 mg/dL were considered diabetic and selected for further studies [58]. The treatment groups were administered either metformin (150 mg/kg) or phytocompound (300 mg/kg) via oral gavage. Based on the findings from the pilot study, specific dosages of 80 mg/kg STZ, 150 mg/kg metformin, and 300 mg/kg phytocompound were selected for the current study. The experimental mice (Table 1) were grouped into five with 15 mice in each group as detailed in Table 1.

No side effects or toxicity were observed in any of the animal groups. All the animal groups were fed a normal chow diet and water throughout this study. All animals used in the experiment were from the F1 generation. The pups from the experimental groups were weaned 21 days after birth. They were then housed and provided with a standard chow diet and water. After two months following a behavioral study, the pups were sacrificed. All the protocols were sanctioned (AWC-23-0055, 28 March 2023) by the Ethical Committee CLAMC (The Center for Laboratory Animal Medicine and Care) in The University of Texas Health Science Center at Houston.

### 4.3. Metabolic Measurements

The body weight and blood glucose levels of the animals were recorded from GD Days 0–18 every 3 days. The body weight was measured using a top-loaded weighing balance (Dr. Trust, New York, NY, USA), and the blood glucose was measured using an Accu-Check active glucose glucometer kit, USA.

### 4.4. Oral Glucose Tolerance Test (OGTT)

At the end of the experimental week, the oral glucose tolerance test (OGTT) was performed by administering 2 g/kg glucose via oral gavage after a 4 h fast. Then, blood glucose concentrations were analyzed using a glucometer at baseline and after glucose administration (30, 60, 90, and 120 min). The results were expressed in mg/dL against recorded time [58].

### 4.5. Tissue Collection

At the end of this study, on Day 19, blood was collected from each mouse, and then they were sacrificed. Blood samples were centrifuged for 15 min at 12,000× *g* at 4 °C to obtain serum and stored at −80 °C until use. Organs, including the brain, pancreas, and gut, were quickly removed and dissected on ice. The gut was collected before perfusion (using PBS); the pancreas and the brain of the mice were removed immediately after perfusion (during sacrifice). For subsequent immunohistochemical analyses, one-half of each organ was immersed and fixed in a 32% formalin solution for 24 h, then stored in 70% alcohol. The other half of the organs were snap-frozen in liquid nitrogen and stored at −80 °C for PCR analysis.

### 4.6. mRNA Gene Expression in the Pancreas

To quantify relative mRNA expression levels of pro-inflammatory cytokines, interleukin (IL)-IL-6, IL-4, and interferon (IFN)-γ RNA was extracted from pancreas tissue samples using the miRNeasy^®^ mini kit (QIAGEN, Montreal, ON, Canada). One µg of RNA was reverse-transcribed to single-stranded cDNA using the RevertAid H minus First Strand cDNA Synthesis Kit (Thermo Fisher, Waltham, MA, USA). Reverse transcriptase real-time (RT) PCR was performed using the Quant Studio 3 Real-Time PCR system (Applied Biosystems, Waltham, MA, USA). The RT-PCR reaction mix (adjusted with H_2_O to a total volume of 20 µL) contained 1 µL template DNA, 10 µL Power SYBR Green PCR master mix (ABI), and 0.5 µL of the respective primers (10 µM each). The forward and reverse primers used for IFN-γ, IL-4, and IL-6 quantification were described previously [17,18]. Relative mRNA target gene expression levels (Ratio = [(E_target_)^dCPtarget (control-sample)^]/[(E_ref._)^dCPref. (control-sample)^]) were normalized to the housekeeping gene glyceraldehyde 3-phosphate dehydrogenase (GAPDH) and used as a reference. Subsequently, the healthy pregnant control group’s pancreatic tissue cytokine was set to 1.0 and used as the calibrator to identify the relative mRNA fold difference between the healthy control and GD groups with and without metformin or phytocompound.

### 4.7. Gut Content Collection and 16s RNA Gene Sequencing

Microbiota in the intestinal luminal content (ileum, cecum, and feces) samples were collected from mice at the time of tissue harvest and stored in sterile tubes at −80 °C until analyzed. The bacteria taxa in each sample were analyzed by amplifying the V4 variable region of the 16S ribosomal RNA (rRNA) gene using high-throughput sequence analysis (Illumina MiSeq platform; Illumina, San Diego, CA, USA) [59]. Quality-filtered 16S rRNA sequences were clustered into operational taxonomic units (OTUs), with 97% similarity, by closed reference OTU-picking using the UCLUST algorithm and GreenGenes reference database (v13.5) as implemented in Quantitative Insights into Microbial Ecology (QIIME versions 1.6 and 1.7) [60]. Sequences were checked for chimeras using ChimeraSlayer with standard options as implemented in QIIME. Sequences that were not clustered were identified using the Ribosomal Database Project to the lowest possible taxonomic level [61,62]. The data were randomly rarefied to 10,000 sequences per sample before any downstream analysis. ATIMA (Agile Toolkit for Incisive Microbial Analyses), developed by the Alkek Center for Metagenomics and Microbiome Research (CMMR) at Baylor College of Medicine, was used for the analysis and visualization of microbiome data sets. 16S RNA sequencing was performed in a single, large experiment to avoid batch effects. Sample cohorts were collected at different timepoints to increase sample size, and samples were stored at −80 °C until sequencing. A rarefaction depth of 5000 reads was applied to normalize sequencing depth across samples between groups.

### 4.8. Histopathology

The gut was collected before perfusion (using PBS), and the pancreas, liver, and brain of the mice were removed immediately after perfusion (during sacrifice) and fixed in formalin solution (32%). Then, the tissues were dehydrated in graded alcohol concentrations and embedded in paraffin. Sections of the brain (10 µm), pancreas (5 µm), and ileum (5 µm) were prepared and stained with hematoxylin and eosin (H&E) [62,63]. The slides were examined under a Keyence BZ-X810 all-in-one confocal microscope for histological changes.

### 4.9. Immunohistochemistry

Immunohistochemistry was performed according to an established protocol [64]. Formalin-fixed, paraffin-embedded 5 μm Ileum sections were subjected to the following staining procedures. Deparaffinized and hydrated sections underwent a heat-induced antigen retrieval procedure using 1 mM EDTA buffer at pH 8.0, followed by washing in 1× PBS. After blocking, sections were incubated overnight with primary antibodies (Table 2), followed by a 60 min incubation with fluorescent secondary immunoglobulins (Table 2). The sections were counterstained with DAPI for 10 min and washed with 1× TBS. Finally, the slides were mounted using Fluoroshield (F6182-20ml, Sigma) for visualization. All immunohistochemistry images were obtained using a Keyence BZ-X810 all-in-one confocal microscope.

### 4.10. Behavioral Analysis

Nest-building, Y-maze, and open-field tests were conducted for each mouse both before and after STZ treatment, metformin, and phytocompound administration. All behavioral tests were administered on the same day. Baseline behavioral data were recorded for all animals before gestation. All behavioral data have been normalized to the baseline behavior. The Y-maze is used to assess and measure the short-term memory and spatial memory of mice. A mouse was placed in the intersection of the three arms of a Y-shaped structure (39.5 × 8.5 × 13 cm^3^) and allowed to move freely through the maze during a 5 min session. Movements were video recorded, and an investigator blinded to the experimental groups analyzed the number of arm entries. The percentage of spontaneous alternation was calculated as [(number of alternations)/(total arm entries − 2)] × 100 [65]. The open-field test is a simple sensorimotor test used to determine the general activity levels, exploration habits, and gross locomotor activity in mice models. A mouse was placed in a squared arena (40 cm per side) and allowed to explore for a 20 min session. Movements were video recorded, and the velocity, distance moved, time spent in the center and borders of the arena, and the frequency of visits to these areas were automatically calculated by the Ethovision XT software (version 17.5) [24]. The nest-building test was conducted to evaluate hippocampal function, assess mood and behavior, and test for movement disorders. The mice were moved into individual cages with a cotton pad (50 × 50 mm^2^). After 18 h, each nest was recorded and scored on a scale of 1–5 by a blinded investigator according to the established criteria [66]. These criteria include scores for the shape of the nest and the amount of material used.

### 4.11. Statistics

Data were tested for normal distribution using the Kolmogorov–Smirnov test. Normally distributed data are presented as means with the standard error, while the medians with their range are given for non-normally distributed data. The significance of differences between the healthy pregnant (Preg) control group compared with the GD-Preg, Met-Preg, and Phyto-Preg groups were analyzed using the one-way analysis of variance test for normally distributed data (or) the Kruskal–Wallis test for non-normally distributed data, followed by either Bonferroni or Tukey’s multiple comparisons post hoc tests. Differences between the groups were considered statistically significant at * *p* < 0.05, ** *p* < 0.01, and *** *p* < 0.001. Prism 10.0 software (Graph Pad Software, Inc., La Jolla, CA, USA) for Windows was used for data presentation and data analysis. All experiments were performed by an investigator blinded to treatments and groups. Differences in Phyla in the gut microbiota of pregnant mice were analyzed using the unweighted UniFrac distance and plotted in a principal coordinates analysis. The UniFrac distance is a metric that evaluates the branch length shared by microbiota under different treatment conditions when mapped onto a common phylogenetic tree [67,68].

## 5. Conclusions

Overall, the results highlighted the potential of *A. nodiflora* as a natural and sustainable alternative to conventional treatments for GD. This green leafy vegetable (GLV) is evident for its higher bioactive compounds, including dietary fiber, polyphenolic antioxidants, fatty acids, vitamins, and amino acids. Beyond providing essential nutrition, these GLVs offer generous health benefits, reduce the risks of cardiovascular disease, cancer, metabolic dysfunctions, and neurocognitive deficits, and improve gut health. Integrating GLVs into the diet, combined with modern healthcare approaches, could help develop sustainable plant-based treatments that improve maternal and fetal outcomes during gestational diabetic pregnancies. Further clinical research is evident to validate these findings and advance the use of *A. nodiflora* in GD management.

## Figures and Tables

**Table 1:** Experimental animal groups.

	Animal Groups	IP	Oral
		Gestational Day 7–11	Gestational Day 13–17
Group I	Non-pregnant Control Group (NPC)	-	-
Group II	Pregnant Control Group (PC)	Citrate Buffer saline	H_2_O
Group III	Gestational Diabetic Group (GD)	80 mg/kg STZ in citrate buffer, pH 4.5	H_2_O
Group IV	Metformin Treatment Group (MT)	80 mg/kg STZ in citrate buffer, pH 4.5	150 mg/kg metformin in H_2_O
Group V	Phytocompound Treatment Group (PT)	80 mg/kg STZ in citrate buffer, pH 4.5	300 mg/kg plant metabolite in H_2_O

H_2_O—water; STZ—Streptozotocin; mg/kg—milligram per kilogram; IP—intraperitoneal.

**Table 2:** List of the antibodies.

Name of the Antibodies	Catalogue No	Company’s Name	Dilution Factor
Ecad	ab11512	Abcam (Cambridge, UK)	1:500
GLUT2	66889-1-IG150UL	Thermo Fisher Scientific	1:300
GLUT3	MA5-32697	Thermo Fisher Scientific	1:300
GFAP	sc-33673	Santa Cruz Biotechnology (Dallas, TX, USA)	1:500
Donkey anti-Rabbit IgG (H + L) Highly Cross-Adsorbed Secondary Antibody, Alexa Fluor™ 647	A-31573	Thermo Fisher Scientific	1:1000
Goat Anti-Mouse IgG H&L (Alexa Fluor^®^ 488)	ab150113	Abcam	1:1000

## Data Availability

The bacterial sequencing data set will be made available upon request. All raw data sets were uploaded in figshare (not publicly available yet) DOI: 10.6084/m9.figshare.28187873.
